# Predicting gene ontology annotations of orphan GWAS genes using protein-protein interactions

**DOI:** 10.1186/1748-7188-9-10

**Published:** 2014-04-03

**Authors:** Usha Kuppuswamy, Seshan Ananthasubramanian, Yanli Wang, Narayanaswamy Balakrishnan, Madhavi K Ganapathiraju

**Affiliations:** 1 Department of Biomedical Informatics and Intelligent Systems Program, University of Pittsburgh, Pittsburgh, PA 15213, USA; 2 Intelligent Systems Program, University of Pittsburgh, 5607 Baum Boulevard, Suite 501 (DBMI), Pittsburgh, PA 15213, USA; 3 Supercomputer Education and Research Centre, Indian Institute of Science, Bangalore 560012, India

**Keywords:** GWAS, GO annotation, Prediction, Function, Protein-protein interactions, Precision-recall curves

## Abstract

**Background:**

The number of genome-wide association studies (GWAS) has increased rapidly in the past couple of years, resulting in the identification of genes associated with different diseases. The next step in translating these findings into biomedically useful information is to find out the mechanism of the action of these genes. However, GWAS studies often implicate genes whose functions are currently unknown; for example, MYEOV, ANKLE1, TMEM45B and ORAOV1 are found to be associated with breast cancer, but their molecular function is unknown.

**Results:**

We carried out Bayesian inference of Gene Ontology (GO) term annotations of genes by employing the directed acyclic graph structure of GO and the network of protein-protein interactions (PPIs). The approach is designed based on the fact that two proteins that interact biophysically would be in physical proximity of each other, would possess complementary molecular function, and play role in related biological processes. Predicted GO terms were ranked according to their relative association scores and the approach was evaluated quantitatively by plotting the precision versus recall values and F-scores (the harmonic mean of precision and recall) versus varying thresholds. Precisions of ~58% and ~ 40% for localization and functions respectively of proteins were determined at a threshold of ~30 (top 30 GO terms in the ranked list). Comparison with function prediction based on semantic similarity among nodes in an ontology and incorporation of those similarities in a k-nearest neighbor classifier confirmed that our results compared favorably.

**Conclusions:**

This approach was applied to predict the cellular component and molecular function GO terms of all human proteins that have interacting partners possessing at least one known GO annotation. The list of predictions is available at http://severus.dbmi.pitt.edu/engo/GOPRED.html. We present the algorithm, evaluations and the results of the computational predictions, especially for genes identified in GWAS studies to be associated with diseases, which are of translational interest.

## Background

Analysis of genetic variations within a population can reveal the genetic predisposition of an individual’s susceptibility to various diseases. Unlike monogenic Mendelian diseases, multifactorial diseases result from combinations of variations in several genes. Therefore, the effect of individual genes on disease-susceptibility is negligibly small, and this necessitates the identification of common genetic variants at multiple loci and their statistical interactions [1], which is challenging [2]. With the development of DNA array technology, a powerful metho4dology has emerged for systematically carrying out genome wide association studies (GWAS). These studies are capable of determining the role of common genetic variants in diseases without the need for establishing the identities of the causal relations [3]. GWAS studies provide a mapping between genetic factors and diseases by drawing comparisons in the genotype of variants between disease cases and controls. These studies are unbiased by current scientific knowledge about individual genes, as they explore genome regions with unknown biological relevance. Therefore, these studies often uncover several genes of unknown functions possibly participating in hitherto unknown biological pathways [4].

A catalog of GWAS studies is maintained by National Human Genome Research Institute (NHGRI) (http://www.genome.gov/gwastudies/) [5]. From this catalog, it may be seen that the number of GWAS studies and the coverage of traits and diseases under these studies are increasing rapidly. As of July 2012 1309 publications have reported GWAS results on 674 traits or diseases (http://www.genome.gov/gwastudies, accessed 2012-July-17). The number of publications increased to 1628 reporting 848 traits (accessed 2013-June-13). The first replicable results from these studies mapped a variation in complement factor H gene to age related macular degeneration [6]. In a GWAS study published in 2007 [7], the following number of association signals reflecting disease susceptibility effects were found: 1 in bipolar disorder, 1 in coronary artery disease, 9 in Crohn’s disease, 3 in rheumatoid arthritis, 7 in type 1 diabetes and 3 in type 2 diabetes. Another GWAS study related to quantitative trait published in 2010 revealed hundreds of genetic variants in 180 loci affecting adult height, a well-known polygenic trait [8].

Though extensive work has been carried out to identify the common genetic variants through GWAS, the exact mechanism of the action and role of these genes are yet to be discovered. Three major areas that need to be explored for understanding the genetic basis of disease susceptibility are [9]: (i) identification of new loci with the genetic variants, (ii) determining the precise position of causal variants and their phenotypic consequences, and (iii) discovery of functional mechanisms of loci and variants, which is the focus of this work.

Here forward we refer to genes identified through GWAS studies in relation to any disease or trait as *GWAS genes.* Lack of knowledge about the functionality of genes/proteins is an impediment to translating the knowledge gained from GWAS studies into clinically or biologically relevant insights. Experimental methods developed to address this issue of protein function prediction focus on the biological knowledge associated with genes and proteins such as gene sequences, protein structure and conformation [10], but these are low throughput in nature [11]. Determining gene/protein functions through experiments is resource intensive, and often the hypotheses for experimentation are not readily visible. It is our goal to develop a computational method to predict the localization, and molecular function of the GWAS genes.

Knowledge based computational techniques for function prediction rely on functional classification schemes [12] such as Functional Catalogue (FunCat) [13], Clusters of Orthologous Groups (COGs) [14], and Gene Ontology (GO) [15], among which GO is the most commonly used annotation system. GO is a controlled vocabulary, which is used to describe the localization, function or the biological process, associated with a gene. All terms in this ontology can be categorized into these three categories, namely cellular component (CC), molecular function (MF) and biological process (BP).

Several previously published algorithms predict the function of a gene from its sequence, protein interaction networks, gene expression data and phylogenetic data [16-19] (see [11] for an extensive survey on different protein function methods and types of datasets).

One of the sequence-based algorithms called protein function prediction (PFP) extracts the GO terms that are closely related to those that are extracted by direct sequence homology matches in a PSI-BLAST search, and scores the GO annotations based on their frequency of occurrence in the retrieved sequences; this way, the predicted GO terms represent functional domains shared by the retrieved proteins. Two of the applications of PFP predictions to large-scale datasets include uncharacterized sequences in several genomes and the *Plasmodium falciparum* (Malaria) PPI network. The performance of PFP was assessed by measuring sequence coverage, annotation specificity and annotation sensitivity. Several other sequence based methods developed include GoFigure [20] and GOtcha [21], which score the GO terms exploiting the hierarchy of the GO DAG structure. In GoFigure, GO database and Saccharomyces Genome Database (SGD) were used. For evaluation, all the genes in SGD were analyzed and SGD was removed from the list of searched databases. The obtained output was then compared to those present in the SGD. GoFigure performs a homology search based on the input DNA or protein sequences and constructs a graph from the extracted GO terms. In Gotcha, databases are searched to obtain sequences similar to query sequence. The processed search results provide pairwise matches with associated R scores (R score = max [-log_10_ (*E*), o], E = Expectancy score for pair), which are then added to the total score for each GO term of the match sequence. The entire graph up to the root node is assigned an R score. The datasets used include the sequences for Malaria, Fruit fly, yeast, human etc. The technique was evaluated by a seven-fold cross validation by comparing the predictions with those provided by the curators of the respective genome sequence consortia.

Lack of completely annotated sequences in the available databases limits the prediction of annotations based on sequence similarity leading to development of other methods which make use of semantic similarity measures for function prediction. The method developed by Pandey et.al, improves on the standard classification-based function prediction algorithms by incorporating the GO DAG structure information into the k-nearest neighbor classifier and uses Lin’s measure for evaluating the semantic similarity between the nodes in the ontology [22]. The information in the neighborhood of proteins being tested for a target class was enhanced using the substantial semantic similarity that exists between the target class and its several neighbors. The results from experiments carried out on an array of datasets showed that the incorporation of the GO DAG structure leads to more accurate predictions. Other methods that use semantic similarity measures include function prediction algorithms proposed by Tao et al. [23] and Tedder et al. [24]. The algorithm by Tao et al. uses information theory-based semantic similarity (ITSS) approach in combination with the GO DAG structure to predict functions of sparsely annotated GO terms. A K nearest -neighbor algorithm along with ITSS measure was used to assign new edges to the concept nodes in the sparse ontology networks. Precision and recall of 90% and 36% respectively for sparsely annotated networks were achieved using a 10 fold cross-validation. In an algorithm called PAGODA (Protein Assignment by Gene Ontology Data Associations), semantic similarity measure is used to group genes into functional clusters, and then a Bayesian classifier is employed for term enrichment by assessing whether a pair of interacting genes belongs to a functional cluster [24]. In this study, eight different *Plasmodium falciparum* datasets were studied. Interaction data for *P. falciparum* was downloaded from the IntAct database. The method was evaluated on all the genes that have GO annotation using a leave-one-out cross validation for each GO term.

Other function prediction algorithms commonly used include methods to extract information from protein-protein interaction networks. Network based approaches may be classified into direct annotations and module assisted schemes [25]. In the direct annotation methods, a protein is assigned the most frequently occurring function among direct interacting partners to the function of the candidate gene (majority rule assignment) or, the assignment was based on a correlation score amongst all possible function pairs of direct interacting partners. However, in the module-assisted methods, the protein networks are first clustered into modules with similar functionality followed by annotation of modules based on the known function of its members.

There have been many computational approaches to GO annotation prediction using the *majority assignment* principle mentioned above. Markov Random Fields [26], Integrated probabilistic models [27], and other Graphical based models [28] have been proposed which make use of the *functional similarity* between interacting proteins. The method developed by Nabieva E. et al. [28] is based on network flow and integrates both network topology and locality measures. Each protein annotated with a function is treated as a source from which “function flows” to other proteins in the network over a period of time. At the end of this period, each unannotated protein is assigned an association score for that function based on the amount of functional flow it received during the fixed period and its location in the PPI network.

Contrary to the majority rule assignment, there have been many methods proposed which make use of GO term associations of *indirect neighbors* (level 1) of a protein in an interaction network. The underlying observation behind this idea was that a protein may not have the same function as its direct interacting partner, but may be similar to its neighbors-of-neighbors [29]. For example, cortistatin (CORT) interacts directly with somatostatin receptors (SSTR), but it is functionally similar to somatostatin (SST), which is a neighbor-of-neighbor to CORTs [30]. Advancing this reasoning, the concept of *shared-neighborhood* has been proposed which identifies the set of interacting proteins that are common to two proteins to identify the confidence of an interaction that can be possible between the pair of proteins [31]. This approach can be extended to the *n*^th^ neighbor in an interaction network, where the *n*^th^ neighbor of a protein can be reached through at-most n direct physical interactions. The protein under consideration is assigned the function that had the highest χ^2^ value among functions of the corresponding *n*-neighbors [32]. While these approaches for function assignment benefit from the information about PPIs, they do not fully exploit the relationships of GO terms among themselves as given by the directed acyclic graph (DAG) structure of the ontology.

One of the approaches that has not been explored in literature is the use of probabilistic approach to predict functions of unknown genes using GO-DAG structure and protein-protein interactions. We propose a probabilistic approach for prediction of hitherto unknown functions of GWAS genes by capturing the complementarity of the GO terms between interacting partners as well as the relations of GO terms in the DAG. The complementarity of GO terms (i.e. the tendency of a pair of GO terms to be associated with each of two interacting proteins) may be computed from the GO annotations of protein pairs in the overall protein-protein interaction network [15].

We compared our approach to a randomized PPI network constructed using the same number of interactions as the original PPI network. To evaluate the ranked lists generated in prediction, we derived the precision recall curves at different threshold values, F-score metric was used to determine the prediction accuracies. Precision indicates the fraction of predicted terms that are relevant and recall determines the fraction of relevant terms that are predicted. Results from this comparison indicate that our method gives higher precision rates for CC and MF categories of 58% (threshold = 33), and 40% (threshold = 28) respectively compared to precision rates of 30% and 18% respectively (at the same thresholds) for CC and MF predictions generated using randomized PPI networks.

Comparison with a function prediction approach based on evaluation of semantic similarity among nodes in an ontology and incorporation of those similarities in a k-nearest neighbor classifier showed that the results from our approach compared favorably.

## Results and discussion

### Approach for GO term prediction and enrichment

We use Bayesian network on the GO DAG to resolve dependencies between GO terms of one protein and those of its interacting partners. Consider protein A that interacts with N proteins p_1_, p_2_, …, p_N_ with a total of k GO terms g_1_, g_2_, …, g_k_. The probability that a GO term *t* is associated with the protein A, given by P (t|g_1_, g_2_, …, g_k_), is predicted using Bayes rule

(1) $$P\left(t\left|g_{1},\;g_{2},\;g_{3},\ldots \ldots g_{k}\right)\right)\propto P\left(\left(g_{1},\;g_{2},\;g_{3},\ldots \ldots ,\;g_{k}\right|t\right)*P\left(t\right)$$

The value *P*(*g*_*1*_, *g*_*2*_, *g*_*3*_, … …, *g*_*k*_|*t*) can be computed as a Bayesian network of GO DAG using its hierarchical structure as:

(2) $$P\left(\left(g_{1},\;g_{2},\;g_{3},\ldots \ldots ,\;g_{k}\right|t\right)=\prod_{i=1}^{k}P\left(g_{i}\left|\mathrm{par}\left(g_{i}\right)\right),t\right)$$

Where par (g_i_ ) denote the set of parent GO terms of the term g_i_ in the GO DAG structure.

The value is *P*(*g*_*i*_|*par*(*g*_*i*_), *t*) computed as follows:

From the training data, the frequencies *f(x,y)* are computed, where *x* and *y* represent GO terms associated with the two proteins of an interacting pair, and *f(x,y)* represents the number of times they occur together amongst all interactions in the training data. Therefore, we have

(3) $$P\left(g_{i}\left|\mathrm{par}\left(g_{i}\right)\right),t\right)=\frac{P\left(g_{i},\left(\mathrm{par}\left(g_{i}\right)\right|t\right)}{P\left(\mathrm{par}\left(g_{i}\right)\left|t\right)\right)}$$

Since g_i_ and par (g_i_) occur together, we get

(4) $$P\left(g_{i},\left(\mathrm{par}\left(g_{i}\right)\right|t\right)=P\left(\left(g_{i}\right|t\right)$$

Substituting (4) in (3):

$$P\left(g_{i}|\mathrm{par}(g_{i}),t\right)=\frac{P\left(g_{i}|t\right)}{P\left(\left(\mathrm{par}(g_{i})|t\right)\right)}=\frac{P\left(g_{i},t\right)}{P\left(\mathrm{par}\left(g_{i}\right),t\right)}=\frac{\mathrm{Count}\left(\text{protein is annotated with}\;g\;\text{and its interacting partner is annotated with}\;t\right)}{\mathrm{Count}\left(\text{potein is annotated with}\;\mathrm{par}\;\left(g\right)\;\text{and its interacting partner is annotated with}\;t\right)}=\frac{f\left(g,t\right)}{f\left(\mathrm{par}\left(g\right),t\right)}$$

The probability of association of a GO term (from the list of CC and MF GO terms) with a gene of unknown function is computed using the information from the probability of occurrence of pairs of GO terms (includes parent GO terms) obtained from the training dataset of interactions and the probability of individual GO term occurrences.

**Figure 1:**
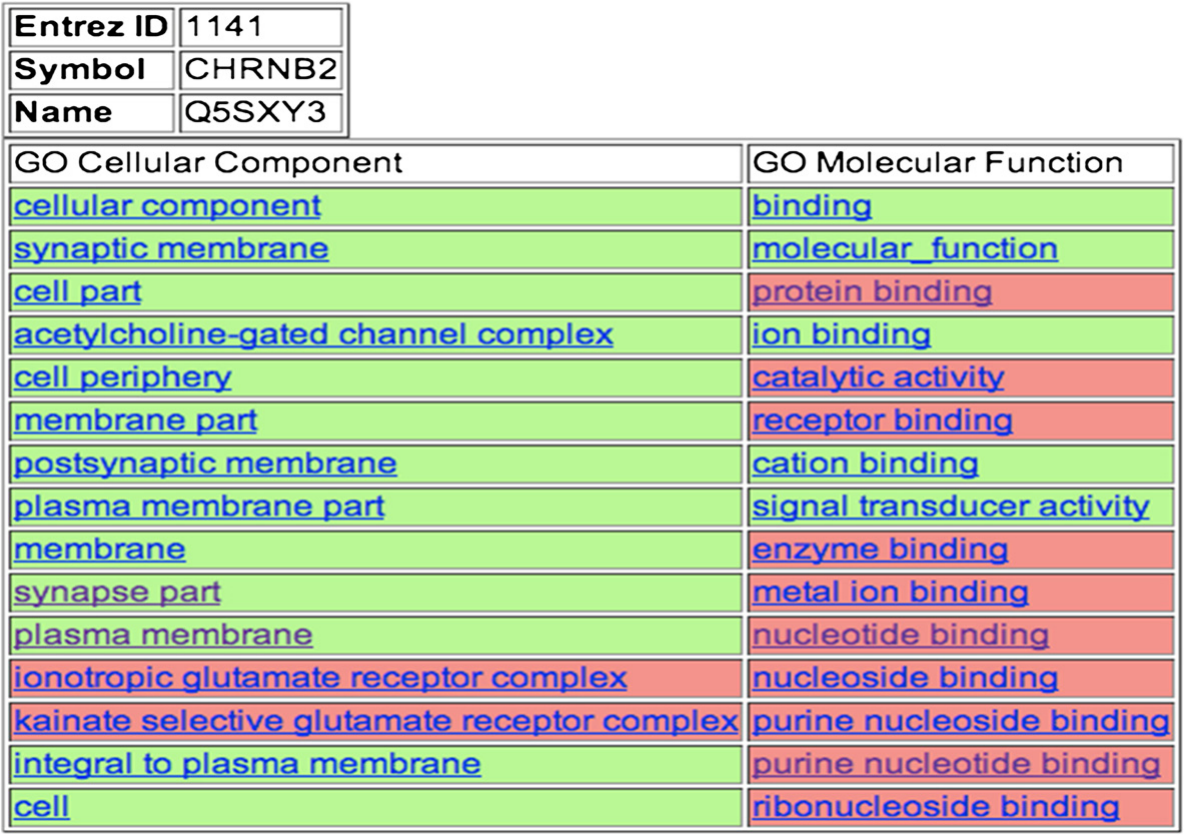
**Rows from color-coded HTML file showing the CC and MF terms for gene CHRNB2: cholinergic receptor, nicotinic, beta 2 (neuronal) (Entrez id: 1141).** GO terms were ranked according to relative association scores for the human genes with atleast one interaction.

**Figure 2:**
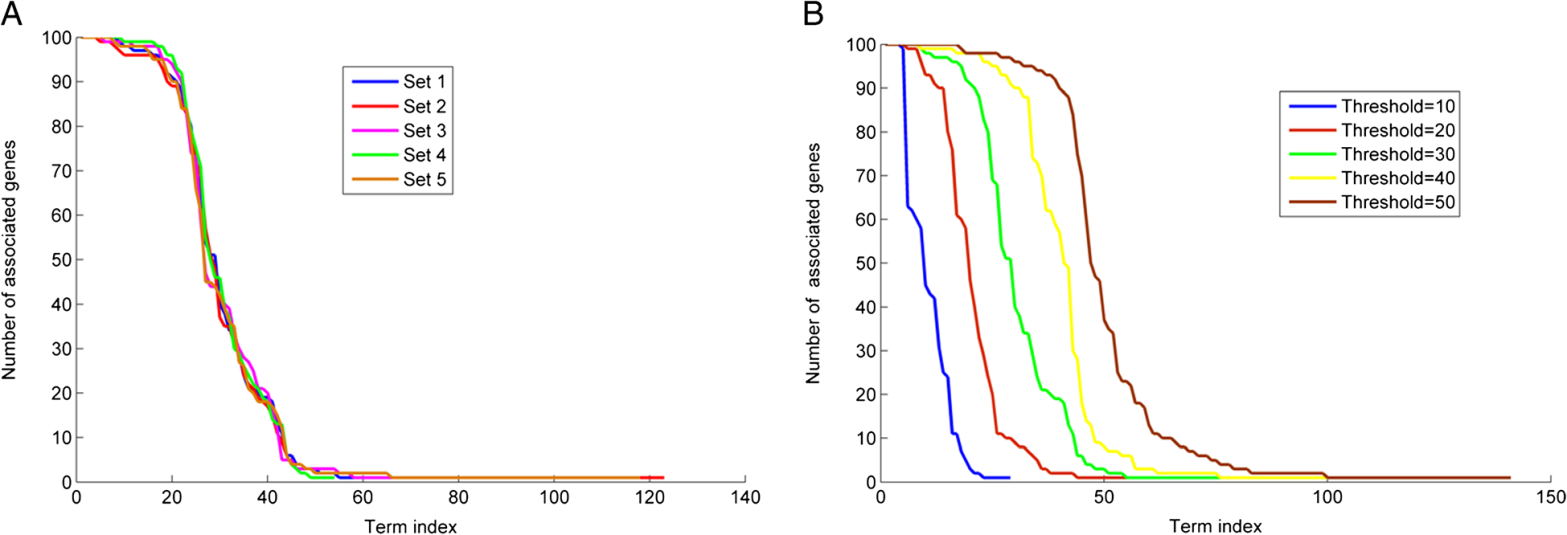
**Evaluation of the specificity of the proposed method for gene annotations predictions.** Figures show the plots of the number of associated genes versus the GO term index **(A)** for all five sets (Set1 to Set5) of 100 genes each at a threshold =30, **(B)** for one set of 100 genes at five thresholds=10, 20, 30, 40, 50.

This approach is employed to predict the GO terms of all human proteins that have known GO annotations with at least one interacting partner. In addition, it enriches the genes that already possess GO associations with additional GO terms. The predicted CC and MF GO terms for each gene were sorted based on the association scores given by (1). For each gene, ranked lists of predicted GO terms are displayed separately for CC and MF in HTML files (http://severus.dbmi.pitt.edu/engo/GOPRED.html). A few rows from a color-coded HTML file with the CC and MF terms for a gene (see “link to detailed version” on the gene page) are shown in Figure 1. The cells in the ranked lists are color coded to show which terms are previously known to be associated with the gene (green) and which terms are novel associations (pink). The ranked lists are useful for manual interpretation of the predicted annotations. As the GO DAG structure up to the root nodes were considered when making predictions, general terms were found at the top of the ranked lists because of the increased frequency of their occurrence among genes. To ensure that our algorithm predicted specific terms as well, at the top of the ranked lists, we extracted the number of gene associations per term for MF category under two circumstances (1) using a threshold of 30 for five sets of 100 genes each and (2) for a given set at different thresholds ranging from 10 to 50 in steps of 10. Plots of the number of genes versus GO terms in both the cases (Figure 2A and B) show GO terms with very few genes associated with them. These results indicate that several specific GO terms were predicted in the top 30 of the ranked list; similar trend was seen at lower thresholds as well, indicating the capability of the model to predict specific terms.

### Evaluation of the proposed approach

The HTML files present qualitative insight into the performance of the algorithm. To evaluate the performance quantitatively, we plotted a set of precision-recall curves using datasets that have *a large number of* known GO terms (as an exhaustive list of GO term associations is not known for even a small set of genes) and at least one PPI. These genes were not included in constructing the probabilistic model.

**Figure 3:**
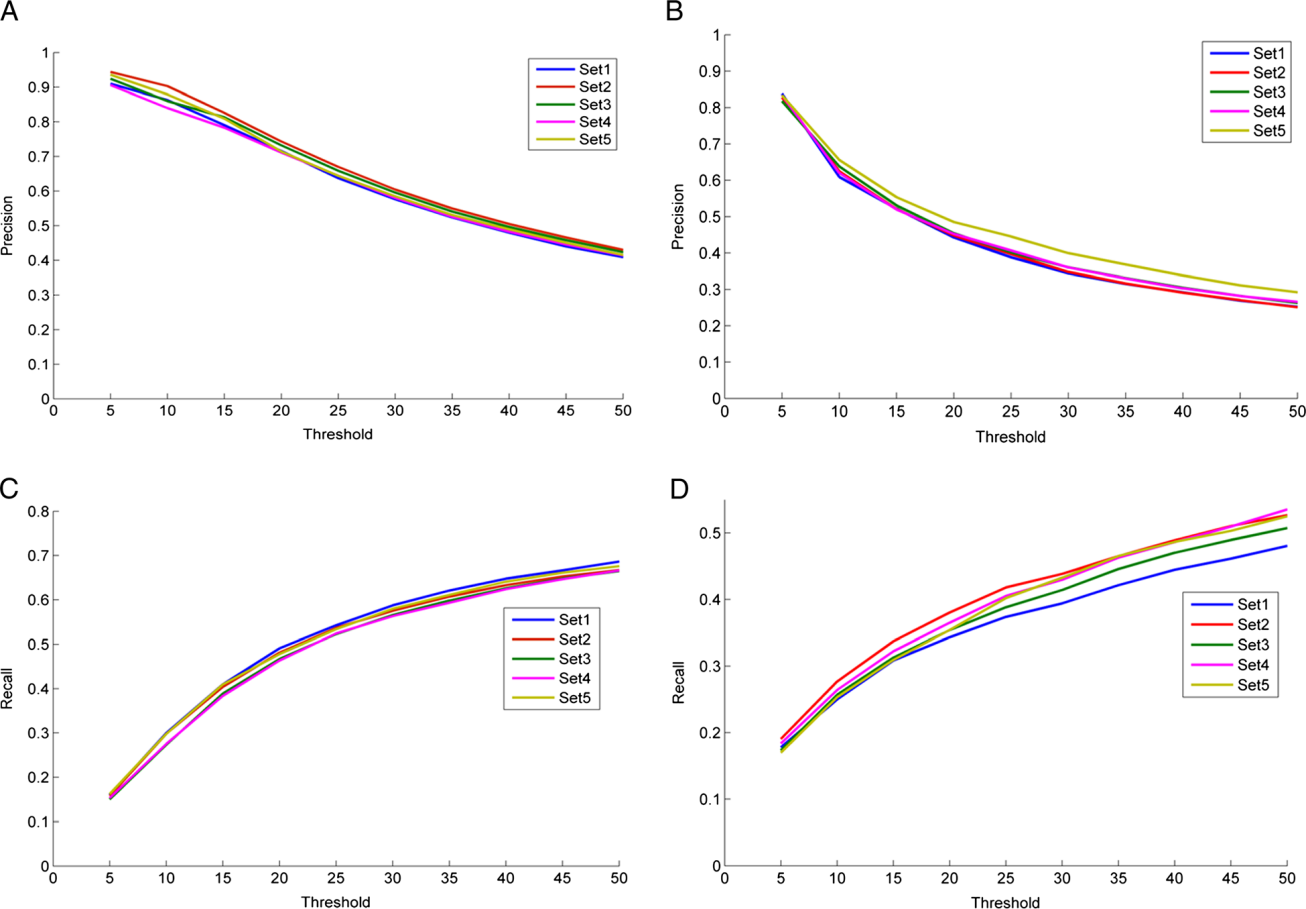
**Evaluation of the proposed method for gene annotations predictions.** Figures in the top panel show the plots of precision versus threshold for 5 sets of 100 genes each for **(A)** CC GO terms and **(B)** MF GO terms. The thresholds ranged from 5 to 50 in steps of 5. Figures in the bottom panel show the plots of recall versus threshold plotted for 5 sets of 100 genes each for **(C)** CC GO terms and **(D)** MF GO terms.

2,500 genes were randomly sampled to create 5 sets of 100 genes each. The GO annotations (CC and MF) for these 5 sets of 100 genes each were predicted and ranked according to their relative association values. **Precision and Recall** values were computed over a range of threshold values (5 to 50, in steps of 5) for each of the 100 genes and averaged over the 100 genes. Precision of the system is defined to be the percentage of correct instances out of the total number of instances predicted, and Recall is defined to be the percentage of correct instances predicted out of the actual number of instances (GO annotations including parents in this case) that are associated with the protein. The average precision and recall curves (for each of the 5 gene sets) plotted against different threshold values are shown in Figures 3A, C for CC and Figures 3B, D for MF. As expected, the precision values were seen to decrease with an increase in the threshold value as GO terms predicted lower down in the ranked lists were irrelevant as opposed to recall that increased with threshold indicating the increase in the total number of relevant terms retrieved.

### Comparison with a randomized PPI network and an existing function prediction algorithm

#### Randomized PPI network

**Figure 4:**
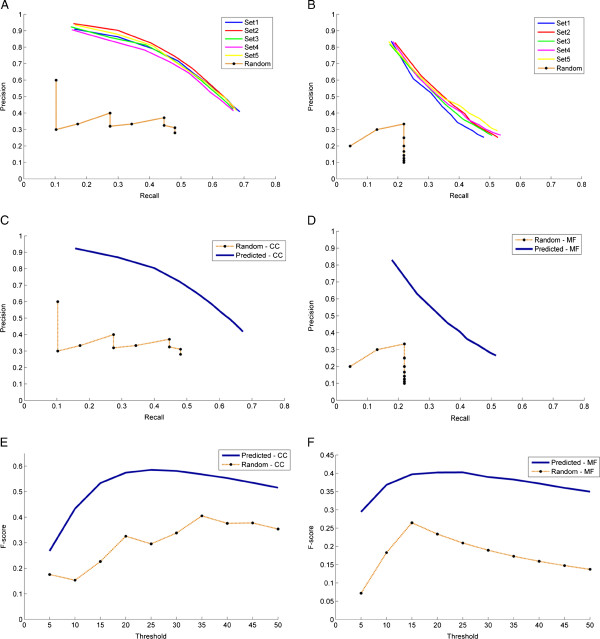
**Comparison of the proposed method with randomized PPI network.** Figures **(A)** and **(B)** show the comparison of precision-recall curves between the proposed method (for each of the five genesets) and a randomized PPI network constructed exploiting the GO DAG structure for **(A)** for CC GO terms and **(B)** MF GO terms. Figures **(C)** and **(D)** show the comparison of average precision-recall curves between the proposed method (averaged over 5 genesets) and a randomized PPI network for **(C)** CC GO terms and **(D)** MF GO terms. Figures **(E)** and **(F)** show the comparison of F-score versus threshold curves, between the proposed method and a randomized PPI network for **(E)** CC GO terms and **(F)** MF GO terms. F-score is calculated as the harmonic mean of precision and recall values.

We compared our method with a randomized PPI network generated as explained in the Methods section. Briefly, the number of edges in a random PPI network was selected to be equal to that of the actual PPI network and the GO DAG structure was exploited to make a fair comparison. Precision - Recall values were computed over a range of threshold values (5 to 50, in steps of 5) for each of the 100 genes and averaged over the 100 genes. The precision**-recall curves plotted** for each of the 5 gene sets are shown in Figure 4A and B for CC and MF respectively. Similar computations were carried out for predictions obtained using a randomized network and compared with the probabilistic results as shown in Figure 4A and B. As seen from the figures, it is clear that the probabilistic method performs better than a randomized network. Precision - Recall curves (averaged over 5 gene sets) were plotted for CC and MF as shown in Figure 4C and D respectively and compared with randomized network curves. As seen from the figures, the precision was compromised at higher recall values and this trend was seen only in the probabilistic case but not seen in the case of randomized predictions. This shows that in the case of randomized predictions, the precision curve is not monotonic because of the arbitrary incidence of predictions in the ranked list of GO terms.

The optimum operating point on the precision recall curves is selected based on the principle of equal error rate, where the precision equals recall. From the precision recall curves for CC, the optimal operating point is computed to be at a threshold of 33 (top 5% of the entire list of CC terms) where precision and recall values equal ~58% compared to a precision/recall of ~30% in the case of random network. In the case of MF, a threshold 28 was chosen with a precision/recall of ~40% compared to ~18% in the case of random network.

F-score measures were derived from the precision recall curves to determine accuracy. The F-score (harmonic mean of precision and recall) at different thresholds were plotted as seen in Figure 4E and F. F-score of about ~58% and ~40% at a threshold of 33 and 28 were obtained for CC and MF respectively.

We compared our approach with a well-established algorithm by Pandey et al. [22]. The algorithm evaluates the semantic similarity between nodes in ontology and incorporates these similarities into k-nearest neighbor classifier. The Mnaimneh gene expression data set [33] was used to generate a detailed list of ranked predictions by the label similarity-incorporated kNN classifiers. 138 BP terms were considered for evaluation. The gene list (1,622 genes) and the 138 BP classes from the same dataset were considered and predictions were carried out using our approach. As our approach results in a ranked list of GO terms as opposed to a ranked list of genes obtained in [22], we created an inverse mapping of GO terms to genes. To obtain the mapping we carried out a per GO term evaluation and identified the genes associated with each GO term. It should be noted that since our approach assigns every GO term to each gene in a ranked fashion (ranked by relative p-value), a threshold of 30 in each ranked list was chosen while obtaining the inverse mapping. This implies that a gene is assumed to be associated with a GO term if the predicted rank of the GO term for that gene is between 1 and 30. The resulting inverse map is a non-ranked list of genes for each GO term, as the relative p-values in our approach are not comparable across genes. However, since the GO term was predicted for those genes within top 30 of the ranked list in our results, all the genes associated with the GO term in the inverse map are likely to have strong association with the term. As the results from Pandey et al.‘s approach is a ranked list of genes for each GO term as opposed to a non-ranked inverse map obtained from our approach, a semi-quantitative measure was developed for comparison. Using this measure, top 20 predicted genes associated with 5 GO terms (with high to low AUC scores) in Pandey et al.’s results were checked for presence in our inverse map results for the same GO terms. It is observed that (Table 1) most of the genes are present in the inverse map. On an average about 18 out of top 20 genes predicted by Pandey et al. are seen to be present in our inverse map for each GO term. This implies that for each GO term, the top predicted genes by Pandey et al. are also likely to be predicted by our approach with a high degree of confidence.

**Table 1:** **Semi-quantitative comparison of probabilistic approach with standard function prediction approach**[22]**using Mnaimneh dataset**

**GO term**	**Fraction of genes correctly predicted by probabilistic approach**	**Gene names (Yeast)**
GO:0008213	18/20	YLR333C, YOR182C, YGR162W, YHR021C, YMR282C, YJL189W, YLR287C-A, YJR056C, YLR455W, YLR185W, YNL313C, YDL002C, YNL132W, YMR031C, YFR032C-A, YNL162W, YML017W, YEL054C
GO:0001510	18/20	YDR161W, YHR052W, YGR162W, YIL091C, YDR101C, YDL063C, YLR009W, YIL096C, YJR032W, YCR016W, YLR287C , YKL078W, YGR071C, YOL077C, YPL226W, YOR361C, YGR173W, YPL193W
GO:0018193	17/20	YOR182C, YHR021C, YJL189W, YLR287C-A, YNL313C, YLR185W, YNL162W, YDL075W, YJL136C, YMR282C, YHR141C, YOL098C, YGR034W, YLR406C, YGR162W, YFR032C-A, YIL069C
GO:0050790	18/20	YGR162W, YLR455W, YNL031C, YMR124W, YLR019W, YGL140C, YEL025C, YMR031C, YBR079C, YFR016C, YJL084C, YBL002W, YJR056C, YDR334W, YPL282C, YNL301C, YMR144W, YLR419W, YKL219W
GO:0009966	18/20	YHR155W, YJL084C, YDR520C, YHL004W, YER033C, YNL289W, YKR104W, YKL209C, YBR071W, YGR162W, YOR077W, YIR016W, YHL029C, YGR054W, YDL123W, YCR030C, YOR166C, YNL208W

Table shows the fraction of the genes correctly predicted using the probabilistic approach. The dataset consisted of 1622 yeast genes and 138 BP GO terms.

### GO term prediction for orphan GWAS genes

We are particularly interested in finding the GO term associations for the genes that are found to be associated to diseases/traits through GWAS.

### Availability of annotations for GWAS genes prior to prediction

**Figure 5:**
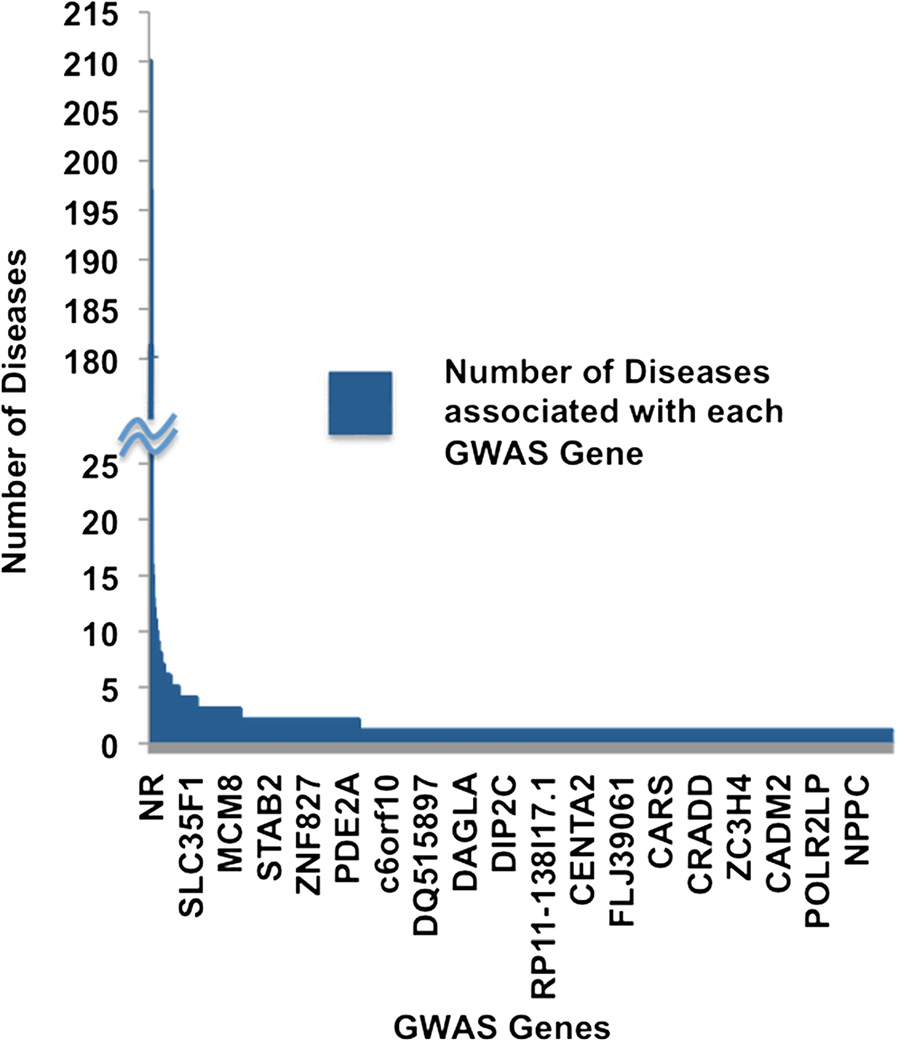
**Plot representing the number of diseases associated with GWAS genes*.*** Individual GWAS genes are plotted on x-axis and the numbers of diseases they are associated with are on y-axis. 4,485 GWAS genes are arranged in descending order of the number of disease/trait associations.

**Figure 6:**
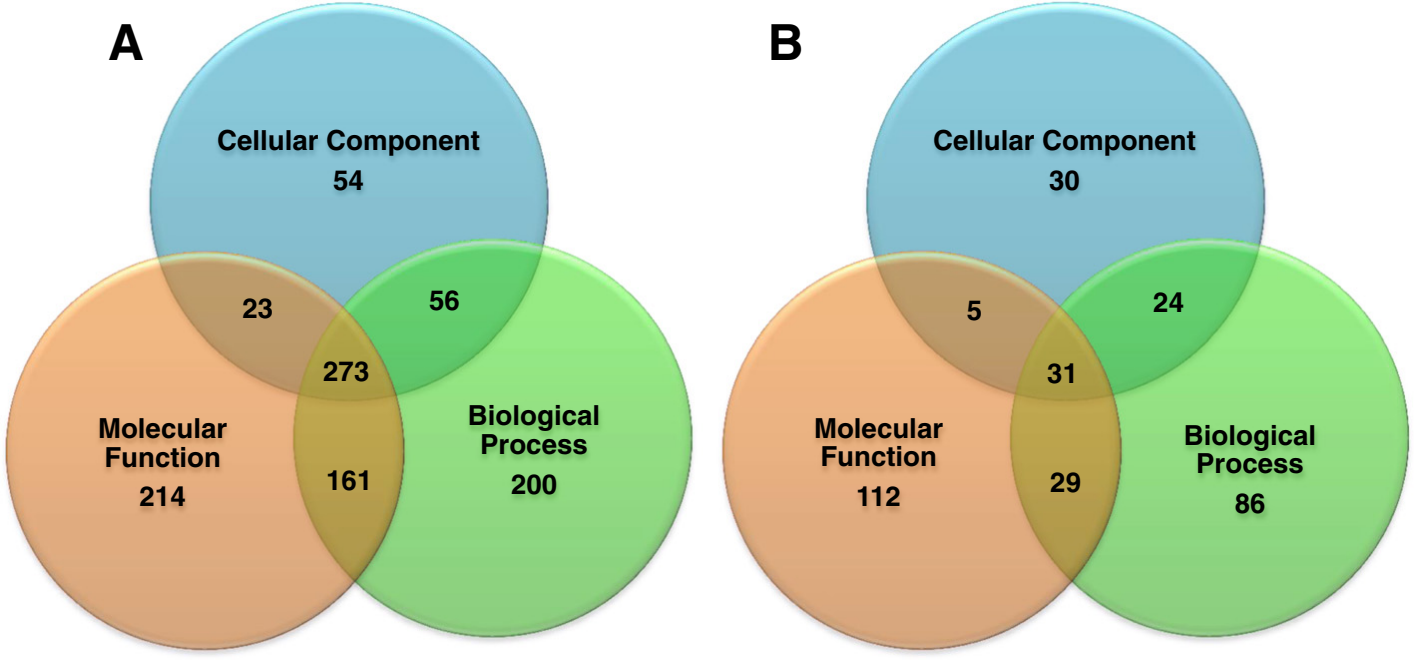
**Distribution of GWAS genes among GO categories: Cellular Component, Biological Process and Molecular Function. (A)** Venn diagram showing the distribution of all identified GWAS genes; about 273 genes were identified with no CC, MF and BP components. **(B)** Venn diagram showing the distribution of GWAS genes listed in the human protein-protein interactions downloaded from HPRD website. About 31 genes were identified with no CC, MF and BP components.

We obtained the gene-disease associations from the GWAS catalog (accessed 2012-July-17). Two of the 4,485 GWAS genes are found to be associated with more than 180 diseases and the remaining 4,483 genes are associated from 1 to 26 diseases each (Figure 5). The distribution for GWAS genes with unknown function is shown in Figure 6A. Out of the 4,483 genes, 54 genes are found to contain no information about GO CC terms, 214 genes have no information about MF terms and about 200 genes have no information about BP terms. 273 genes are found to not have any of the three terms. As our approach relies on GO terms of interacting partners, in this study GWAS genes with unknown functions but with at least one known interaction were considered. With this constraint, the dataset contained about 31 genes without known GO terms as shown in Figure 6B. GO annotations of all GWAS genes are predicted using the proposed approach leading to novel GO term annotations when none were previously known, or GO term enrichment when some were known.

**Figure 7:**
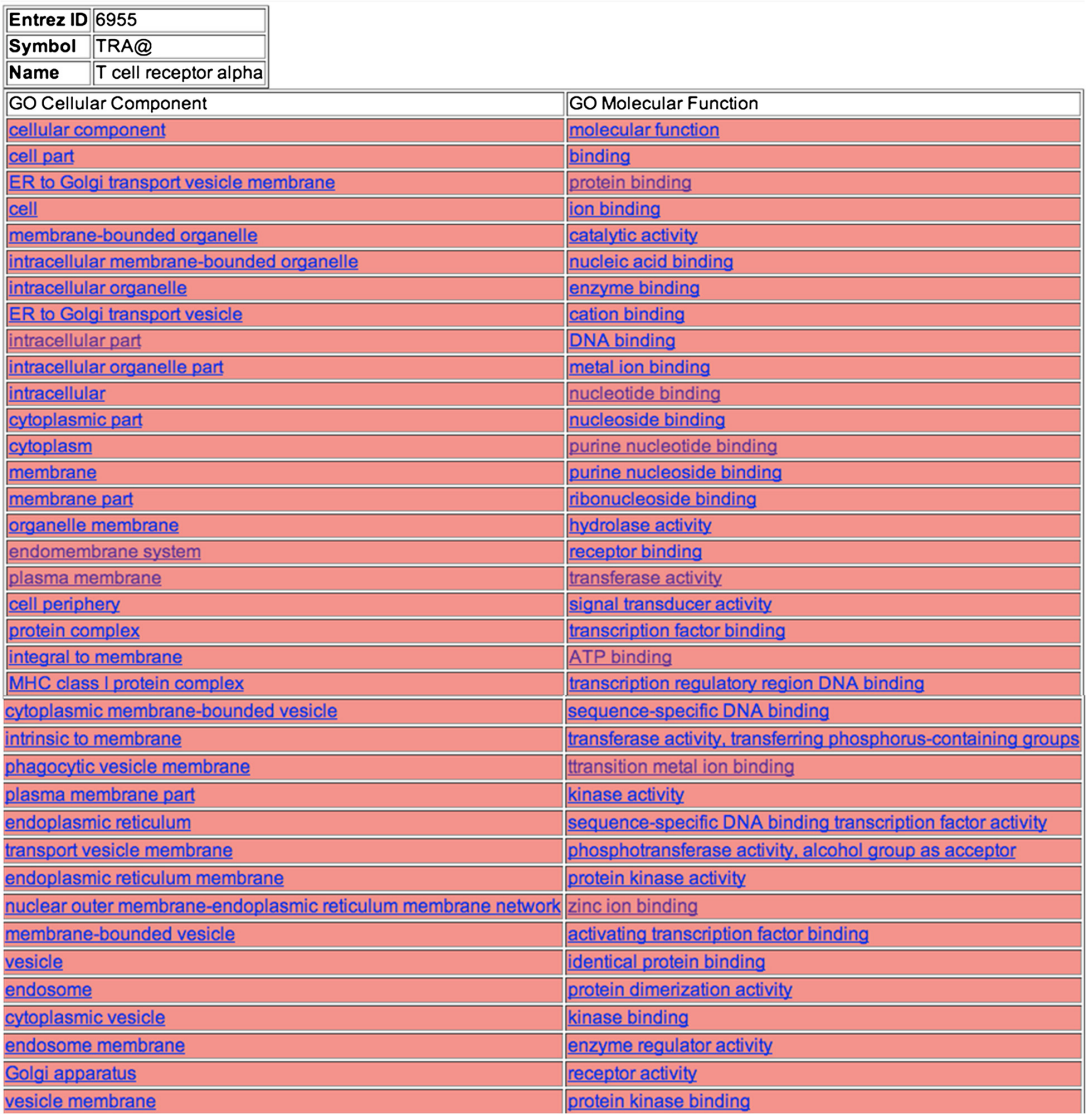
**Rows from color-coded HTML file showing the CC and MF terms for GWAS gene TRA@ T cell receptor alpha locus (Entrez id: 6955) associated with Narcolepsy.** GO terms were ranked according to relative association scores for the human GWAS genes with atleast one known interaction**.**

CC and MF GO terms were predicted for the list of 3 GWAS genes with unknown GO terms (‘orphan genes’) using the probabilistic algorithm (color coded ranked lists are given at http://severus.dbmi.pitt.edu/engo/gogwas.html). An example of a ranked list is shown in Figure 7. Given the knowledge of the association of GWAS genes to diseases, we carried out some intuitive evaluation to see if the predictions seem relevant. We chose a couple of genes from the list of GWAS genes and looked at their predicted MF and CC GO terms.

### SYN2

The *SYN2* gene encodes synapsin II, which encompasses the alternatively spliced proteins synapsin IIa and IIb and belongs to the family of synaptic vesicle-associated phosphoproteins. Synapsin II is known to localize to the cytoplasmic surface of synaptic vesicles in the axonal terminal of presynaptic neurons [34]. These neuronal phosphoproteins function to modulate neurotransmitter release [35] and are implicated in many neuropsychiatric diseases such as schizophrenia. Interestingly, SYN2 has been implicated in Type II diabetes by GWAS analysis.

#### *SYN2*’s role in Type II diabetes

Type II diabetes is a chronic condition characterized by elevated blood glucose level due a combination of resistance to insulin by certain tissues and deficient insulin secretion by the pancreatic β Langerhan cells [36]. Insulin is an anabolic hormone responsible for glucose transport and storage as well as glycogen, triglyceride and protein synthesis: essential processes in tissues such as liver, adipose and muscle that become resistant to the effects of insulin in Type II diabetes. The secretion of insulin in response to glucose (rather than the basal release) occurs in a biphasic manner [37]. The first phase encompasses the immediate exocytosis of insulin from granules that are readily docked, and the second phase requires mobilization of the larger pool of insulin granules in reserve, which is a slower and more sustained process. In type II diabetes, a defect in the early, fast phase of insulin release is more prominently observed [38]. Therefore, a defect in either the signaling pathway involved in or the structural component of vesicle release of insulin granules must drive the pathogenesis of Type II diabetes. Looking at the predictions for *SYN2,* then, terms such as **membrane-bounded organelle, synapse**, **vesicle** or **cytoplasmic membrane-bound vesicle** suggest its localization to synaptic vesicles. Furthermore, *SYN2*, like its better-characterized counterpart *SYN1*, contains phosphorylation sites for calcium-calmodulin dependent protein kinase [39]. The existence of these sites in concordance with the predictions **protein serine/threonine kinase activity** suggests that such signal transduction pathways could be involved in regulating insulin release through the activity of *SYN2*. Therefore, it could be hypothesized that *SYN2* variants could potentially play a role in the pathogenesis of Type II diabetes through the defect in modulation of insulin granule exocytosis, particularly during the first phase of insulin release.

Similarly the predicted terms **cell projection, neuron projection, synapse** in addition to **vesicle** or **membrane-bounded vesicles** confirm its role in neurodegenerative diseases such as schizophrenia that is associated with the dysfunction of synaptic transmission due to various molecules such as synapsins. The concentration of these molecules is reduced in the brain of schizophrenics and therefore affects the neurotransmitter release that immediately precedes vesicle fusion [40].

### TRA

TRA gene encodes the T-cell receptor alpha chain [34] and is found to be associated with Narcolepsy that is a neurological disorder characterized by sleepiness or inability to regulate sleep cycles [41]. Narcolepsy has been linked to polymorphisms in genes encoding the T cell receptor alpha chain [42]. Hypocretin, a neurotransmitter plays an important role in sleep-wake cycles [43]. Deficiency in hypocretin producing neurons in the brain results in this disorder that is usually accompanied by cataplexy (emotionally triggered loss of muscle tone) [41].

In individuals predisposed to narcolepsy, one of the common factors involves the Human Leukocute Antigen (HLA) complex found on chromosome 6. HLA complexes are major histocompatibility complexes (MHC) that are cell-surface molecules mediating interactions of immune cells. HLA complexes corresponding to MHC-1 class, display proteins produced within the cells to the T cells that then eliminate the infected cells. The variations in these HLA genes result in the decrease of the neurons producing hypocretin due to an increase in autoimmune response to these proteins [41]. Looking into the predictions for TRA gene, terms such as **protein complex, MHC class 1 protein complex, and phagocytic vesicle membrane** suggest the role of auto-immunity in narcolepsy.

The immune response of T cells is characterized by a series of biochemical events that involve several enzymes, co-receptors and transcription factors. The T-cell receptor alpha is presented with the antigens bound to MHC-1 molecules through an endogenous pathway. In the endogenous pathway, the peptide fragments are transported to the lumen of the endoplasmic reticulum (ER) through transporter proteins. The peptide fragments then bind to the MHC-1 protein and the Golgi apparatus transports this complex to the surface of the cell where it is recognized by the T cells [44]. Predicted GO terms including **enzyme activity, transcription factor binding, ER to Golgi transport vesicle membrane, ER to Golgi transport vesicle, endoplasmic reticulum, endoplasmic reticulum membrane or nuclear outer membrane-endoplasmic reticulum membrane network** advocate the system of antigen presentation to T cells.

## Conclusions

We developed a probabilistic method to predict the functions and localizations of genes, based on PPIs. The method associates a GO term to a gene with probability values that are calculated based on the frequency of occurrence of the GO term with the GO terms of interacting partners of the gene. The ontology structure of the annotations is also considered while making predictions. Exploitation of the GO DAG structure results in general terms being predicted at the top of the ranked lists. However, our algorithm also predicts more specific terms within the top 5% of the total list of GO terms considered, without compromising precision. Future work would entail incorporation of some form of normalization based on the frequency of occurrence of GO terms to eliminate the general terms. Systematic comparison with the randomly generated PPI networks (with GO DAG) shows better prediction accuracies with our approach. **Comparison with** an existing function prediction algorithm resulted in similar predictions demonstrating the efficiency of our approach to predict functions of genes.

The approach was used to predict the CC and MF GO terms of orphan GWAS genes. Similarly, the BP terms for GWAS genes can also be predicted using this approach.

An extension of the project would involve comparison of our approach with random PPI networks without GO DAG, to access the relative importance of GO DAG versus PPI in prediction accuracies of genes. There are several functions in nature yet to be determined which are likely to be associated with the existing annotated proteins. Our algorithm predicts several novel functions; however these need to be experimentally verified further to confirm the validity of their association with a corresponding gene.

Overall, this work illustrates the effectiveness of using the knowledge of protein interactions in conjunction with GO DAG structure for predicting annotations of genes with unknown function. The approach shows significant promise by predicting specific GO terms for the GWAS genes that are of translational interest.

## Materials and methods

### Datasets

The Gene Ontology Consortium (http://www.geneontology.org) presents a controlled vocabulary for annotating proteins by categorizing the protein features into three general types: cellular component (CC), biological process (BP) and molecular function (MF). The terms in the ontology are arranged in the form of a directed acyclic graph (DAG) in which the GO terms are divided into increasingly specific details further away from root. A directed edge from GO term *x* to GO term y confers the relationship that *y* is an instance of *x,* meaning that when a gene or protein is associated with *y*, it would also be associated with *x*. This association continues up to the root of the tree. The consortium also annotates genes with the ontology terms by curating literature.

A list of annotated human-genes is obtained from the gene ontology website (accessed July 2012). A list of about 48,419 known protein-protein interactions (accessed July 2012) is obtained from the Human Protein Reference Database (http://www.hprd.org) and the BioGRID (http://thebiogrid.org/). There are about 18,005 genes that are annotated with GO terms and about 5,092 that have at least one known interaction. From these genes, five datasets of 100 genes each were created having highest number of GO terms and atleast one interaction. The given annotations of genes are extended to explicitly include the ancestors. For evaluation of our algorithm, the developed method is compared to a random PPI network constructed exploiting the GO DAG structure for a fair comparison. To construct the random network, the number of interactions (edges) is fixed to be equal to the number of interactions in the actual network. The enriched terms are ranked in descending order of significance. Finally, the algorithm is employed to predict the functions of orphan GWAS proteins with unknown GO terms. Computations for modeling and prediction are carried out for CC and MF separately by considering only those GO terms that correspond to these categories.

#### Dataset of GWAS genes with unknown functions

The list of GWAS genes obtained from the GWAS catalog was parsed. Of these, the genes with at least one interaction but not having known GO CC or MF terms were collected. The GO terms of these genes were then predicted using the information from protein-protein interaction network. Genes with only CC, only BP and only MF missing were identified along with the genes with GO terms of two missing categories (i.e. both CC and MF, CC and BP, CC and MF).

### Evaluation of the performance of the algorithm

The idea is to carry out quantitative evaluation of the algorithm performance by plotting the precision and recall values vs threshold for all 5 datasets. The known GO term annotations are removed from the test sets and these are predicted using the data from the training set. For each category, the predicted GO terms are then compared with original annotations to determine the number of correctly predicted annotations. For each gene, the percentage of predicted GO terms that are relevant (precision) and the percentage of relevant GO terms that are predicted (recall) are computed at different threshold values ranging from 5 to 50 (in steps of 5). The precision and recall values are averaged over 100 genes and are plotted against threshold values for each gene set. Precision versus recall curves for each gene set were also plotted.

#### Comparison of our method with random PPI networks

The performance of the algorithm is compared to random PPI network generated by exploiting the GO DAG structure. The random network is constructed to have random interactions between proteins but the number of edges is maintained equal to the actual PPI network. The precision-recall values along with F-scores (harmonic mean of precision and recall) were computed for both the actual network (averaged over 5 datasets) and random network at 10 different thresholds 5 to 50 in steps of 5. Plots of precision versus recall and F-scores versus threshold were plotted for both cases.

## Availability of data

The predictions for all the human proteins (with atleast one known protein-protein interaction) are available at http://severus.dbmi.pitt.edu/engo/GOPRED.html. The ranked list of prediction for GWAS genes is available at http://severus.dbmi.pitt.edu/engo/gogwas.html. The website shows the GO terms for CC and MF separately, ranked in the descending order of their association probability values. The source code and the input data files may be downloaded from http://severus.dbmi.pitt.edu/engo/.

## Abbreviations

GO: Gene ontology; GWAS: Genome wide association studies; PPI: Protein-protein interactions; CC: Cellular component; MF: Molecular function; DAG: Directed acyclic graph.

## Competing interests

The authors declare that they have no competing interests.

## Authors’ contributions

The algorithm has been developed and implemented by SA, UK with some contributions by YW and evaluated by UK under the guidance of MKG and conceptualization by NB. The manuscript has been prepared by UK, MG and reviewed and approved by all authors.
